# Cerebrovascular function and its association with systemic artery function and stiffness in older adults with and without mild cognitive impairment

**DOI:** 10.1007/s00421-022-04956-w

**Published:** 2022-05-06

**Authors:** Tom G. Bailey, Timo Klein, Annelise L. Meneses, Kayla B. Stefanidis, Stefanie Ruediger, Daniel J. Green, Tim Stuckenschneider, Stefan Schneider, Christopher D. Askew

**Affiliations:** 1 VasoActive Research Group, School of Health, University of the Sunshine Coast, 90 Sippy Downs Drive, Sippy Downs, QLD 4556 Australia; 2 Physiology and Ultrasound Laboratory in Science and Exercise, Centre for Research on Exercise, Physical Activity and Health, School of Human Movement and Nutrition Sciences, The University of Queensland, Brisbane, QLD Australia; 3 Institute of Movement and Neurosciences, German Sport University, Cologne, Germany; 4 Institute of Sport Science, University of Rostock, Rostock, Germany; 5 School of Human Sciences, Faculty of Science, The University of Western Australia, Crawley, WA Australia; 6 Sunshine Coast Health Institute, Sunshine Coast Hospital and Health Service, Birtinya, QLD Australia

**Keywords:** Cognition, Dementia, Pulse wave velocity, Cerebral blood flow, Pulsatility index, Flow-mediated dilation

## Abstract

**Purpose:**

Our aim was to compare cerebrovascular and systemic vascular function between older adults with and without mild cognitive impairment (MCI), and to determine which measures of vascular function best predict the presence of MCI.

**Methods:**

In 41 adults with MCI and 33 adults without MCI (control) we compared middle cerebral artery velocity (MCAv) and cerebrovascular pulsatility index (PI) at rest, cerebrovascular reactivity to CO_2_, and responsiveness to changes in blood pressure (%∆MCAv/%∆MAP). Systemic vascular function was assessed by flow-mediated dilation (FMD) and stiffness by pulse wave velocity (PWV).

**Results:**

Cerebrovascular PI was higher in MCI compared with control (mean ± SD: 1.17 ± 0.27 vs. 1.04 ± 0.21), and MCI exhibited a lower %∆MCAv/%∆MAP (1.26 ± 0.44 vs. 1.50 ± 0.55%). Absolute (*p* = 0.76) and relative cerebrovascular reactivity to CO_2_ (*p* = 0.34) was similar between MCI and control. When age was included as a covariate the significant difference in cerebral PI between groups was lost. PWV was higher (13.2 ± 2.2 vs. 11.3 ± 2.5 m s^−1^) and FMD% (4.41 ± 1.70 vs. 5.43 ± 2.15%) was lower in MCI compared with control. FMD% was positively associated with PI across the cohort. Logistic regression analysis indicated that FMD and PWV significantly discriminated between MCI and controls, independent of age, whereas the inclusion of cerebrovascular measures did not improve the predictive accuracy of the model.

**Conclusion:**

These findings raise the possibility that early changes in systemic vascular stiffness and endothelial function may contribute to altered cerebrovascular haemodynamics and impaired cognitive function, and present potential targets for prevention and treatment strategies in people with MCI.

**Supplementary Information:**

The online version contains supplementary material available at 10.1007/s00421-022-04956-w.

## Introduction

The prevalence of dementia is increasing and is expected to directly affect 74 million adults worldwide by 2030 (WHO 2017). Prevention of dementia and cognitive impairment in older adults is a major public health challenge, and thus the identification of modifiable risk factors across the spectrum of the disease is needed. The characterisation of adults with mild cognitive impairment (MCI) has been advocated because MCI increases the risk of clinical dementia by up to 10 times (WHO 2017), and some people demonstrate a capacity to restore cognitive function (Aretouli et al. 2010). There is a strong association between cardiovascular risk factors and cognitive decline (Baumgart et al. 2015; Veldsman et al. 2020). However, the association between systemic vascular and cerebrovascular function, and the extent to which these parameters are able to discriminate between adults with and without MCI is not yet fully established.

Using transcranial Doppler (TCD) ultrasonography to investigate the intracranial arteries, there is evidence that the velocity of cerebral blood flow (CBFv) is lower, indicative of chronic hypoperfusion, in adults with MCI (Beishon et al. 2017). However, alterations in cerebrovascular function via assessments of cerebrovascular reactivity to inspired CO_2_ and perturbations in blood pressure, i.e. dynamic autoregulation, are conflicting (Lim et al. 2018; Tarumi et al. 2014; de Heus et al. 2018). There appears to be a disparity between changes in global cerebral blood flow in adults with MCI, and the corresponding measures of cerebrovascular function. Such measures of large cerebral artery blood flow and reactivity do not likely reflect the early cerebral small-vessel and microvascular changes that are reported in MCI (Toth et al. 2017), and this may explain the variance in previous reports (Lim et al. 2018; Shim et al. 2015; Tarumi et al. 2014; de Heus et al. 2018). The brain is characterised by a high-flow, low-resistance vascular network, and the exposure of small vessels to high pulsatile flow is suggested to contribute to downstream microvascular damage (Cooper et al. 2016; Cooper and Mitchell 2016). Increases in cerebral vascular resistance, reflecting a limited ability of the cerebral vessels to damp the pulsatile cerebral blood flow, is a contributing factor to elevations in cerebral pulsatility. There is emerging evidence that measures of cerebral pulsatility are associated with MCI (Shim et al. 2015; Roher et al. 2011; Vinciguerra et al. 2019) and may therefore provide a more sensitive marker of the associated cerebrovascular alterations than global cerebral blood flow and intracranial artery reactivity.

A growing body of research also highlights the association between systemic vascular function and brain health in ageing adults. For example, evidence from cross-sectional studies demonstrate that reductions in reactive hyperaemia and flow-mediated dilation (FMD), and elevated pulse wave velocity (PWV), are associated with poor cognitive performance and steeper declines in cognition with age (Venturelli et al. 2018; Vendemiale et al. 2013b; Iulita et al. 2018). Recent longitudinal evidence in a large cohort of older adults showed that elevated aortic stiffness, measured as PWV, was an independent predictor of the development of MCI (Pase et al. 2016). Indeed, there is a potentially significant role of systemic haemodynamic pulsatility on the structure and function of the brain (Avolio et al. 2018). Elevated arterial stiffness impairs the pressure buffering capacity of the aorta and carotid arteries, leading to an increase in pressure pulsatility (Iulita et al. 2018). It is suggested that central artery stiffening contributes to the propagation of high pulsatile pressures, particularly towards the low-resistance peripheral vascular beds such as in the brain (O'Rourke and Safar 2005; Avolio et al. 2018). For this reason, elevated cerebrovascular pulsatility may not only reflect cerebrovascular resistance, but might be due to high aortic stiffness. Given the emerging importance of cerebral pulsatility for brain health (Roher et al. 2011; Hughes et al. 2018; van Sloten et al. 2015), there is a need to better understand the potential central determinants, including aortic stiffness, central pulse wave parameters, and systemic vascular function, in adults displaying mild cognitive impairment.

A better understanding of the relationship between cerebrovascular and systemic haemodynamics during the early stages of cognitive decline could highlight potential detection and/or treatment strategies. Therefore, the aim of this study was to compare measures of cerebrovascular and systemic vascular function, including cerebrovascular reactivity, cerebral pulsatility, aortic stiffness and systemic endothelial function, and to identify which combination of measures best discriminates between older adults with and without MCI. Our primary hypothesis was that the cerebral pulsatility index would be elevated in people with MCI compared with control participants, and would be associated with aortic stiffness measured using pulse wave velocity.

## Methods

### Participants

This study included a comparison of adults with MCI (*n* = 41) and control participants (*n* = 33). Those with MCI were recruited through the NeuroExercise project (Devenney et al. 2017) at the German Sport University and were invited to undergo a series of additional cerebrovascular and cardiovascular assessments that form the basis of this cross-sectional investigation. Healthy adults of a similar age were recruited as control participants through local community advertisements. The study conformed to the *Declaration of Helsinki* (1975) and was approved by the institutional human research ethics committees. Participants provided written informed consent and were screened to ensure they met the inclusion criteria prior to participation.

MCI was classified according to established clinical criteria (Albert et al. 2011b), including the presence of memory decline without dementia (Clinical Dementia rating global score of 0.5), and a Montreal cognitive assessment (MoCA) score of 18–25 (Nasreddine et al. 2005). All MCI participants underwent cognitive assessment with a neuropsychology dementia specialist. Control participants did not report any memory impairments during an initial screening for subjective cognitive decline based on the framework by Jessen et al. (2014). Prospective participants were asked: (1) if they have any difficulty remembering routine tasks, (2) remembering things that happened recently, (3) whether others had expressed concerns regarding their memory, and (4) if they feel their memory is any worse than that of others of a similar age. Control participants achieved a score ≥ 26 on the MoCA during pre-study screening. MCI and control participants were aged between 65 and 85 years, had no history of prior cardiovascular events (i.e. no previous myocardial infarction or stroke), and prospective participants with hypertension (> 160/100 mmHg) that was not being treated were excluded.

### Study overview

After screening, all participants attended the laboratory on a single occasion to undergo a series of cerebrovascular and systemic vascular assessments. Participants refrained from alcohol and exercise for 24 h and caffeine for 12 h before the session and continued to take prescribed medication. Participants were fitted with instruments to measure middle cerebral artery blood flow velocity (MCAv), mean arterial blood pressure (MAP), end-tidal carbon dioxide (P_ET_CO_2_), and heart rate (HR) which were recorded throughout the study session. After resting supine measurements were obtained, cerebrovascular CO_2_ reactivity was assessed using a breath-hold test. Participants then performed a repeated stand-to-sit test for the determination of cerebral pressure-flow responsiveness. Baroreflex sensitivity was also assessed during the repeated stand-to-sit procedure. Following this, participants underwent a further 15 min of supine rest prior to assessments of peripheral and central blood pressure, wave reflection characteristics, and carotid-femoral PWV. Finally, participants underwent a test of brachial artery FMD as a measure of systemic endothelial function. All assessments were conducted in a quiet, temperature controlled (23 ± 0.5 °C) laboratory.

### Resting cerebrovascular measures and cerebrovascular pulsatility index

Resting measurements were collected during a 5-min period of supine rest before the cerebrovascular function test. Cerebrovascular conductance was calculated as MCAv relative to MAP. Cerebrovascular pulsatility index (PI) was calculated using Gosling and King’s equation [PI = (*V*_s_ − *V*_d_)/*V*_mean_] (Gosling and King 1974), where *V*_s_ is the systolic velocity, *V*_d_ the diastolic velocity and V_mean_ the mean velocity.

### Breath hold test—cerebrovascular reactivity to CO_2_

Cerebrovascular reactivity was assessed as the MCAv response to changes in P_ET_CO_2_ that were induced with a repeated breath-hold test while in the supine position. The breath-hold test is a validated procedure to assess cerebrovascular function (Tancredi and Hoge 2013). After a period of paced breathing, participants held their breath for 20 s. A metronome was used as a guide for paced breathing at a rate of 16 breaths per min for 30 s before the next breath-hold started. Participants were instructed to give a small, forced exhalation at the end of each breath-hold. For each breath-hold manoeuvre, the increase in P_ET_CO_2_ (∆P_ET_CO_2_) was calculated by subtracting the average over the last two breaths before the breath-hold from the peak P_ET_CO_2_ response immediately after the breath-hold. The first two breath-hold manoeuvres were used to familiarise participants with the procedure (Tancredi and Hoge 2013). Breath-holds were analysed separately and a total of six breath-hold responses per person were averaged for comparison between groups (Tancredi and Hoge 2013; Murphy et al. 2012). Cerebrovascular reactivity was calculated as the increase in MCAv relative to the corresponding increase in P_ET_CO_2_ as absolute (∆MCAv/∆P_ET_CO_2_) and relative (%∆MCAv/∆P_ET_CO_2_) responses.

### Stand-to-sit test: cerebral pressure-flow responsiveness and baroreflex sensitivity

The repeated stand-to-sit transition test consisted of 13 stand-to-sit transitions in a 5-min period at a frequency of 0.05 Hz (10 s in the standing position and 10 s in the seated position). This same protocol has previously been used in older adults (van Beek et al. 2008; van Beek et al. 2010; Oudegeest-Sander et al. 2014; den Abeelen et al. 2014; Klein et al. 2020). The angle of the left knee was continuously measured with a bipolar sensor (Goniometer, MLTS700, ADInstrument, Bella Vista, NSW, Australia) to enable the alignment of data with each period of standing and sitting. During each repeated stand-to-sit transition, maximum values during the sit phase, and minimum (nadir) values during the stand phase, were identified for MCAv, MAP and P_ET_CO_2_. Whereas for HR responses, maximum values were identified during the stand phase and minimum values during each sit phase. For each transition, the response of each variable was calculated using the delta (sit − stand). The transition delta responses were also expressed as a percentage of the stand phase values (%∆), and the ratio of %∆MCAv/%∆MAP was calculated for each transition as previously reported (Brassard et al. 2017; Klein et al. 2020). Baroreflex sensitivity was calculated as the change from stand to sit in R-R interval relative to the change in systolic blood pressure (Xing et al. 2017). For each variable, responses during each of the 13 stand-to-sit transitions were averaged for further comparison between the groups.

### Instrumentation and measurements for cerebrovascular assessments

MCAv was measured continuously using transcranial Doppler ultrasonography (TCD) where a 2 Hz probe was placed over the right temporal window and fixed at a constant angle with a customised headband (Multigon, Neurovision, Elmsford, N.Y., USA). The MCAv signal was identified according to standardized criteria guided by signal depth, velocity and wave characteristics (Aaslid et al. 1982; Willie et al. 2011), which remained constant for the testing session. If a right MCAv signal could not be clearly obtained, the left MCAv was used (*n* = 7). Heart rate was monitored by ECG and blood pressure was measured continuously at the left middle- or index-finger using photoplethysmography (Finometer MIDI, Finapres Medical Systems, Amsterdam, The Netherlands). Participants breathed through a leak-free respiratory mask (Hans-Rudolph, Kansas City, MO, USA), and expired air was continuously sampled for the determination of P_ET_CO_2_. All variables were sampled at 1000 Hz and stored (LabChart Pro v7.3.7 and PowerLab, ADInstruments, Bella Vista, NSW, Australia). Time-aligned signals were then resampled to second-by-second (1 Hz) for visual inspection and analysis.

### Blood pressure, wave reflection characteristics and pulse wave velocity

Indices of peripheral and central blood pressure, wave reflection characteristics and pulse wave velocity were measured in the supine position using the SphygmoCor XCEL device (AtCor Medical, West Ryde, NSW, Australia). These tests have previously been described in detail (Perissiou et al. 2018) and were conducted in accordance with standardised guidelines (Townsend et al. 2015). Blood pressure was measured in triplicate on the right arm, with the first assessment discarded and the average of the last two used. Carotid-to-femoral PWV was obtained by measuring the right carotid pulse wave using applanation tonometry, and the femoral pulse wave using a pneumatic cuff (inflated to 80 mmHg) at the right mid-thigh. Once a regular and stable carotid pulse was detected, femoral pulse waves were collected simultaneously by inflation of the thigh cuff. An aortic pressure waveform was generated over 10 cardiac cycles, from which central systolic (cSBP) and diastolic pressures (cDBP), augmentation pressure (AP) and index (AIx) were calculated. Wave separation analysis was applied (SphygmoCor CVMS software, v.9) to determine the aortic forward (*P*_f_) and backward (*P*_b_) pressure waveforms, which were expressed as the wave reflection magnitude (RM; *P*_b_/*P*_f_ × 100). This method assumes a triangular-shaped flow wave approximated from the estimated aortic pressure wave (Westerhof et al. 2006). The *P*_f_ and *P*_b_ pressure waves correspond to the peak and the end of the assumed flow wave, respectively. PWV was calculated as the distance (m) between the carotid and femoral sites multiplied by 0.8, divided by the time delay (s) between the pulse waves (Townsend et al. 2015). PWV was measured in duplicate, with an average recorded. If the measurement difference was > 0.5 m/s, a third measurement was performed and the median taken (Townsend et al. 2015; Huybrechts et al. 2011).

### Brachial artery flow-mediated dilation

Brachial artery FMD was performed in line with technical recommendations described elsewhere (Thijssen et al. 2011; Black et al. 2008) and in accordance with previous methods described by our group (Bailey et al. 2018, 2017). FMD was performed with participants in the supine position, on the right arm with the cuff placed distal to the olecranon process. High-resolution duplex ultrasound with a 12-MHz multi-frequency linear array probe (T3000; Terason, Burlington, MA) was used to image the brachial artery in the distal third of the upper arm and to simultaneously record the longitudinal B-mode image and Doppler blood velocity trace. The Doppler angle of insonation was maintained at 60°. Images were optimised, and the settings (depth, focus position and gain) were maintained during each assessment. Following a 60-s recording period of diameter and velocity, the cuff was rapidly inflated (220 mmHg) and maintained for 5 min (D.E. Hokanson, Bellevue, WA). Diameter and velocity recordings resumed 30 s prior to rapid cuff deflation (< 2 s) and continued for 3 min thereafter. Analysis of brachial artery diameter was performed using custom-designed edge-detection and wall-tracking software, which is largely independent of investigator bias. Detailed descriptions of the analysis approach are described elsewhere (Thijssen et al. 2011; Black et al. 2008). FMD was calculated as [(peak diameter − baseline diameter)/baseline diameter] and expressed as a percent change in vessel diameter. FMD% was also corrected for variability in baseline diameter between groups, and expressed as scaled FMD (Atkinson et al. 2013). From synchronised diameter and velocity data, blood flow (the product of lumen cross-sectional area and Doppler velocity) was calculated at 30 Hz. Shear rate was calculated as 8 times mean blood velocity/vessel diameter (expressed as s^−1^). Our laboratory has previously reported a between and within day coefficient of variation (CV) for FMD% of < 10% (Bailey et al. 2018). Consistent with recommendations for scaling FMD (Atkinson et al. 2013) and to account for the influence of baseline artery diameter (Atkinson 2013), FMD% was also calculated by allometric scaling of logarithmically transformed absolute diameter change [difference between peak artery diameter and baseline diameter (in mm)]. The logged absolute diameter changes were then back-transformed and interpreted in the conventional manner to obtain allometrically scaled FMD (percent diameter change).

### Statistical analysis

Based on the mean effect extracted from a pooled dataset of > 500 MCI and control participants across multiple studies (Roher et al. 2011; Shim et al. 2015; Vinciguerra et al. 2019) this study was powered (alpha 0.05, beta 0.90) to detect a mean difference of 0.22 ± 0.20 in the primary variable of interest (cerebrovascular PI) between MCI and control. Gaussian distribution and homogeneity of variance of the data were confirmed using Shapiro–Wilk and Levene tests. Comparisons of cerebrovascular and systemic vascular function assessments were made between groups (MCI v CON) using independent *t* tests. To account for the potential influence of age on vascular outcomes, an ANCOVA was used to further analyse differences in key outcomes of interest between MCI and CON. Binary hierarchical logistic regression analysis was employed to examine whether the systemic (PWV and FMD) and cerebral (%MCA/%MAP and PI) measures of interest were able to discriminate between MCI and control. Age was entered at Step 1, followed by the systemic and cerebral variables of interest at Step 2. Large confidence intervals were observed for the odds ratio for PI. To address this, PI scores were subsequently categorised as high versus low (with values < 1 coded as low). However, treating PI as a categorical variable did not affect the results and thus raw values are reported.

Pearson’s correlation coefficients were used to examine the relationships between cerebrovascular and systemic function responses in the MCI and control groups. The effect size is reported as low if the value of r is around 0.1, medium if *r* ≥ 0.3, and large if *r* ≥ 0.5 (Cohen 2013). Statistical significance was set at *p* < 0.05*.* Statistical analyses were performed with Statistica 7.1. (StatSoft, Tulsa, USA).

A complete dataset (MCI: *n* = 41, CON: *n* = 33) was available for the analysis of MoCA scores and other participant characteristics, as well as the assessment of central blood pressure and augmentation index. Due to technical difficulties in obtaining reliable MCAv or finger blood pressure signals during the sit-to-stand test, there were seven missing cases from the MCI group (MCI: *n* = 34, CON: *n* = 33); and an additional four missing MCI cases for the cerebrovascular reactivity dataset because of difficulties performing the breath hold manoeuvre (MCI: *n* = 30, CON: *n* = 33). For the assessment of pulse wave velocity there were four missing cases from the MCI group and three missing cases from the CON group (MCI: *n* = 37, CON: *n* = 30) because a reliable 10 s carotid artery pulse wave signal could not be obtained. Flow mediated dilation could not be assessed in two MCI cases due to poor ultrasound image quality (MCI: *n* = 39, CON: *n* = 33). Analyses were carried out with all available data without substitution or imputation for missing cases.

## Results

### Participant characteristics

Participant characteristics are presented in Table 1. Mean age of participants with MCI (75 years) was slightly greater than the control participants (71 years, *p* = 0.001); and the MCI group also had a significantly lower MoCA score (*p* < 0.001). The significant difference in MoCA scores between groups remained when accounting for age (*p* < 0.001). Resting heart rate, peripheral blood pressures, and BMI were all similar between groups. There was a difference in aspirin medication use between MCI and controls (*p* < 0.001), and higher beta blocker use in MCI (*p* = 0.06); but the use of other medications was similar between groups.

**Table 1:** Participant characteristics

Variable	MCI (*n* = 41)	CON (*n* = 33)	*p* value
	Mean ± SD	Mean ± SD	
MoCA score	22 ± 2	27 ± 3	** < 0.001**
Age (years)	75 ± 5	71 ± 5	**0.001**
(Female: Male)	15:26	19:14	
Height (cm)	170 ± 8.9	170 ± 8.4	0.645
Weight (kg)	77 ± 14.4	74 ± 11	0.420
BMI (kg/m^2^)	27.6 ± 7.7	25 ± 3	0.137
HR (bpm)	60.9 ± 9.4	62 ± 10	0.623
SBP (mmHg)	137 ± 18	135 ± 14	0.676
DBP (mmHg)	74 ± 9	76 ± 9	0.358
Comorbidities, *n* (%)			
Hypertension	13 (33)	10 (25)	0.219
Hyperlipidaemia	3 (17)	4 (22)	0.823
Medication, *n* (%)			
Aspirin	18 (44)	2 (6)	** < 0.001**
Beta-Blocker	13 (32)	2 (6)	0.062
Angiotensin receptor blocker	13 (32)	4 (13)	0.340
Calcium Channel blocker	5 (12)	4 (13)	0.106
Statin	10 (24)	4 (13)	0.707

Bold *p* values indicate a significant difference (*p* < 0.05)

*MoCA* Montreal Cognitive Assessment, *BMI* body mass index, *HR* heart rate, *SBP* systolic blood pressure, *DBP* diastolic blood pressure

### Cerebrovascular measures

Cerebrovascular outcomes are presented in Table 2. Cerebrovascular PI was significantly higher in MCI compared to control (*p* = 0.03), but this effect was lost when accounting for age as a covariate (*p* = 0.35). Cerebral pressure-flow responsiveness and cerebrovascular reactivity to CO_2_ are presented in Tables 2 and 3, respectively. Resting MCAv, P_ET_CO_2_ and MAP were not different between MCI and control groups. The MCI group showed a lower %∆MCAv/%∆MAP response than the control group (*p* = 0.048). When accounting for age the difference in the %∆MCAv/%∆MAP was lost (*p* = 0.08).

**Table 2:** Cerebrovascular pulsatility index and average cerebral pressure-flow responsiveness during repeated stand-to-sit transitions in MCI and control groups

Variable	MCI (*n* = 34)	CON (*n* = 33)	*p* value
	Mean ± SD	Mean ± SD	
PI (ratio)	1.17 ± 0.27	1.04 ± 0.21	**0.030**
MCAv—nadir stand (cm s^−1^)	41.43 ± 8.61	46.90 ± 10.68	**0.024**
MCAv—maximum sit (cm s^−1^)	55.45 ± 11.82	60.86 ± 14.43	0.097
MCAv—∆sit-stand (cm s^−1^)	14.03 ± 6.23	13.89 ± 5.58	0.923
MAP—nadir stand (mmHg)	71.80 ± 13.53	77.83 ± 17.75	0.122
MAP—maximum sit (mmHg)	91.29 ± 14.01	96.67 ± 18.41	0.182
MAP—∆sit-stand (mmHg)	19.62 ± 5.76	18.79 ± 6.05	0.568
P_ET_CO_2_—∆sit-stand (mmHg)	2.96 ± 1.11	2.72 ± 1.18	0.425
CO—∆sit-stand (mmHg)	1.10 ± 0.74	0.87 ± 0.81	0.232
BRS—∆sit-stand (mmHg)	3.00 ± 3.28	2.47 ± 3.24	0.508
%MCAv/%MAP (%%)	1.26 ± 0.44	1.50 ± 0.55	**0.048**

Bold *p* values indicate a significant difference (*p* < 0.05)

Data represent the average responses for the repeated stand-to-sit transitions

*PI* pulsatility index, *MCAv* middle cerebral artery flow velocity, *MAP* mean arterial blood pressure, *P*_*ET*_*CO*_*2*_ partial pressure of end-tidal carbon dioxide, *CO* cardiac output, *BRS* baroreflex sensitivity, *MCI* mild cognitive impairment, *CON* control

**Table 3:** Cerebrovascular reactivity in MCI and control groups

Variable	MCI (*n* = 30)	CON (*n* = 33)	*p* value
	Mean ± SD	Mean ± SD	
P_ET_CO_2_—pre (mmHg)	30.72 ± 5.47	29.30 ± 4.51	0.263
P_ET_CO_2_—peak (mmHg)	37.26 ± 4.86	36.73 ± 4.99	0.670
P_ET_CO_2_—∆ (mmHg)	6.54 ± 2.64	7.43 ± 3.09	0.226
MCAv—pre (cm s^−1^)	40.69 ± 10.42	44.59 ± 12.52	0.191
MCAv—peak (cm s^−1^)	46.50 ± 11.56	52.71 ± 13.99	0.064
MCAv—∆ (cm s^−1^)	5.81 ± 2.75	8.12 ± 4.16	**0.014**
MAP—pre (mmHg)	69.91 ± 11.93	72.35 ± 14.94	0.481
MAP—peak (mmHg)	72.96 ± 12.75	76.89 ± 16.60	0.300
MAP—∆ (mmHg)	3.05 ± 5.48	4.55 ± 6.93	0.348
CO^2^ reactivity—(cm s^−1^ mmHg^−1^)	1.31 ± 0.99	1.38 ± 1.05	0.765
CO^2^ reactivity—(% cm s^−1^ mmHg^−1^)	2.41 ± 1.36	2.76 ± 1.51	0.341
CVC reactivity—(∆mmHg^−1^)	0.014 ± 0.015	0.012 ± 0.023	0.761

Bold *p* values indicate a significant difference (*p* < 0.05)

Data represent the average responses of six consecutive breath-holds

*MCAv* middle cerebral artery flow velocity, *MAP* mean arterial blood pressure, *P*_*ET*_*CO*_*2*_ partial pressure of end-tidal carbon dioxide, *CO*_*2*_* reactivity* cerebrovascular reactivity, *CVC* *reactivity* cerebrovascular conductance reactivity, *MCI* mild cognitive impairment, *CON* control

The change in P_ET_CO_2_ was similar between groups during the breath-hold test. While the change in MCAv was significantly higher in control compared to MCI (*p* = 0.014), the peak MCAv also tended to be higher in control compared to MCI, but this did not reach statistical significance (*p* = 0.064). The cerebrovascular conductance (CVC) reactivity to CO_2_ (*p* = 0.761) and relative cerebrovascular reactivity (*p* = 0.341) was similar between MCI and control groups. Findings for cerebrovascular reactivity were unaffected when accounting for age as a covariate (*p* = 0.75).

### Systemic vascular measures

Indices of central blood pressure, wave reflection characteristics, pulse wave velocity and vascular function are presented in Table 4. Similar to the findings for peripheral blood pressure, there were no significant differences in central blood pressure or wave reflection characteristics between MCI and control groups. PWV was significantly higher in MCI compared with control (*p* = 0.002). Brachial artery FMD% was lower in MCI compared to control (*p* = 0.030). When accounting for age as a covariate, the significant differences in both PWV (*p* = 0.004) and FMD% (*p* = 0.02) remained. Across both cerebrovascular and systemic vascular outcomes, we observed similar differences between MCI and control groups when the male and female cohorts were considered separately (Refer to Online Resource: Supplementary Table 1). While some of the group differences were no longer statistically significant, the magnitude of the differences in means were similar to the effects reported in the full cohort.

**Table 4:** Central wave characteristics, arterial stiffness, and vascular function parameters

Variable	MCI (*n* = 41)	CON (*n* = 33)	*p* value
	Mean ± SD	Mean ± SD	
*Central haemodynamic and arterial stiffness indices*			
Systolic pressure—(mmHg)	125.0 ± 15.7	123.8 ± 14.4	0.746
Pulse pressure—(mmHg)	49.6 ± 14.2	46.4 ± 10.8	0.293
Augmentation pressure—(mmHg)	15.0 ± 7.1	13.27 ± 5.7	0.266
AIx75—(%)	22.9 ± 10.8	21.6 ± 9.1	0.585
Pf—(mmHg)	33.0 ± 9.6	30.6 ± 5.7	0.217
Pb—(mmHg)	20.3 ± 5.1	18.4 ± 3.9	0.210
RM—(%)	62 ± 8	61 ± 6	0.662
PWV—(m.s^−1^)	13.2 + 2.2 (*n* = 37)	11.3 ± 2.5 (*n* = 30)	**0.002**

Variable	MCI (*n* = 39)	CON (*n* = 33)	*p* value
	Mean ± SD	Mean ± SD	
*Flow-mediated dilation*			
Baseline diameter—(cm)	0.47 ± 0.07	0.43 ± 0.08	**0.019**
Peak diameter—(cm)	0.49 ± 0.07	0.45 ± 0.08	**0.026**
FMD—(%)	4.41 ± 1.78	5.43 ± 2.15	**0.030**
Scaled FMD—(%)	4.30 ± 1.70	5.27 ± 2.04	**0.030**
Time to peak—(s)	61.5 ± 24.5	52.9 ± 19.3	0.110
SR_AUC_—(10^3^ s^−1^)	13.36 ± 8.19	16.68 ± 8.80	0.103

Bold *p* values indicate a significant difference (*p* < 0.05)

*FMD* flow-mediated dilation, *AIx75* augmentation index normalized to a heart rate of 75 bpm, *Pf* forward pressure wave, *Pb* backward pressure wave, *RM* reflection magnitude, *PWV* pulse wave velocity, SR shear rate, *AUC* area under the curve, *MCI* mild cognitive impairment, *CON* control

### Logistic regression: vascular function measures that predict MCI

#### Model 1: systemic and cerebral outcome measures

Age significantly predicted group membership, accounting for 17.8% of the variance (*χ*^2^(1) = 8.601, *p* = 0.003, Nagelkerke *R*^2^ = 0.178 (Table 5). Adding the systemic and cerebral vascular outcome measures significantly improved the predictive accuracy of the model, accounting for 47.7% of the variance (*χ*^2^(4) = 17.98, *p* = 0.001, Nagelkerke *R*^2^ = 0.477). However, only the systemic measures contributed significantly to the model. Accordingly, a second logistic regression analysis was performed, excluding the cerebral measures.

**Table 5:** Logistic regression analysis of the systemic and cerebral vascular outcomes to predict MCI group membership (Model 1)

	*B*	SE	*p*	OR	95% CI for OR	
Model 1						
Step 1						
Age	0.152	0.056	0.006	1.164	1.044	1.298
Step 2						
Age	0.134	0.071	0.058	1.144	0.996	1.314
FMD	-0.463	0.204	0.023	0.629	0.422	0.939
PWV	0.463	0.185	0.012	1.588	1.106	2.280
%%MCAMAP	-0.956	0.711	0.179	0.384	0.095	1.548
PI	2.787	1.77	0.115	16.23	0.506	520.953

*FMD* flow-mediated dilation, *PWV* pulse wave velocity, *MCAv* middle cerebral artery flow velocity, *MAP* mean arterial blood pressure, *PI* pulsatility index, *OR* odds ratio

#### Model 2: systemic outcome measures only

Including systemic vascular outcomes only (FMD and PWV) accounted for 72.3% of the sample variance (Table 6). For each unit increase in FMD value, the odds of being in the MCI group decreases by 0.650, and for each unit increase in PWV values, the odds of being in the MCI group increases by 1.58 (i.e., by 58%).

**Table 6:** Logistic regression analysis of the systemic vascular outcomes to predict MCI group membership (Model 2)

	*B*	SE	*p*	OR	95% CI for OR	
Model 2						
Step 1						
Age	0.139	0.051	0.007	1.149	1.040	1.271
Step 2						
Age	0.164	0.061	0.007	1.178	1.046	1.327
FMD	− 0.431	0.172	0.012	0.650	0.463	0.911
PWV	0.460	0.170	0.007	1.584	1.136	2.208

*FMD* flow-mediated dilation, *PWV* pulse wave velocity, *MCAv* middle cerebral artery flow velocity, *MAP* mean arterial blood pressure, *PI* pulsatility index, *OR* odds ratio

### Associations between cerebrovascular and systemic vascular outcomes in MCI and control

Correlations between MoCA scores and the cerebral and systemic outcomes are presented as an Online Resource (Supplementary Table 2). MoCA score was associated with FMD in the pooled cohort (*n* = 71, *r* = 0.28, *p* = 0.017). Cerebrovascular PI was associated with systolic blood pressure in the pooled cohort (*n* = 67, *r* = 0.43, *p* < 0.001), and this association was stronger in MCI (*n* = 34, *r* = 0.46, *p* < 0.01) than in control (*n* = 33, *r* = 0.36, *p* < 0.05). Cerebrovascular PI was also negatively associated with diastolic MCAv at rest when examining the pooled cohort (*n* = 67, *r* = − 0.49, *p* < 0.01). These findings are shown as an Online Resource (Supplementary Fig. 1). When considered separately, this association remained significant in control (*n* = 33, *r* = − 0.53, *p* < 0.01) and in MCI (*n* = 34, *r* = − 0.39, *p* = 0.022). We found no significant associations between PWV and cerebrovascular parameters in MCI or control groups (*p* > 0.05). FMD% was positively associated with PI in the pooled cohort (*n* = 67, *r* = 0.28, *p* = 0.022), and this association was strong in MCI (*n* = 34, *r* = 0.56, *p* < 0.001), but not in control (*n* = 33, *r* = 0.23, *p* = 0.19).

## Discussion

This study aimed to compare cerebrovascular function, including cerebral PI, and systemic endothelial function and arterial stiffness between older adults with and without MCI. In the cerebrovasculature, there were negligible differences between groups in resting cerebral blood velocity and cerebrovascular reactivity to changes in carbon dioxide (using a breath-hold test). We observed a higher cerebral PI and lower cerebrovascular responsiveness to changes in blood pressure (stand–sit test) in adults with MCI compared with control participants. For systemic vascular outcomes we observed higher aortic stiffness (PWV) and lower systemic endothelial function (FMD) in adults with MCI compared to control. When controlling for age, the observed differences in cerebral outcomes were lost and only systemic vascular outcomes (FMD and PWV) differed between MCI and control. This suggests that MCI is likely responsible for the differences we observed in systemic vascular outcomes.

Resting cerebral blood velocity (MCAv) was similar between groups in this study. Previous studies have shown that cerebral blood flow gradually decreases across the spectrum of cognitive impairment, particularly when investigations include adults across a wide spectrum of disease severity (Beishon et al. 2017). Our findings support those of De Heus et al. (2018) who reported significantly lower MCAv in adults with established dementia compared to control, but not in MCI (de Heus et al. 2018). One of the primary risk factors for end organ damage, including at the heart, kidneys and brain, is the chronic elevation in blood pressure, and large oscillations in daily blood pressure (Rickards and Tzeng 2014). A strength of our study is that resting blood pressure and heart rate were similar between MCI and control. The MCI group had higher statin and beta-blocker use, which may improve vascular function including FMD (Peller et al. 2015; Ruszkowski et al. 2019; Zhang et al. 2012). Taken together, our findings suggest that resting MCAv alone does not discriminate between adults with and without MCI, and therefore does not provide explicit insight into the impact of early-stage cognitive decline on cerebrovascular control.

Cerebral PI is traditionally interpreted as a measure of downstream cerebrovascular resistance (Fleysher et al. 2018) and is suggested to provide additional prognostic information for small cerebral vessel disease over that of resting blood flow, e.g. MCAv (Kidwell et al. 2001). Previous reports show that cerebral pulsatility is associated with small cerebral vessel disease (Shi et al. 2018) and poor cognitive performance (Lim et al. 2017). Cerebral PI was elevated in adults with MCI in this study compared with control, and values reported were higher than age-expected estimations (Alwatban et al. 2019). Our observations are supported by prior evidence for elevated cerebral PI in adults with cognitive decline (Roher et al. 2011; Anzola et al. 2011). Vinciguerra et al (2019) reported elevated cerebral PI in adults with vascular cognitive impairment who exhibited white matter lesions, compared to adults without cognitive impairment or white matter lesions. While cerebral PI is indicative of increased downstream resistance and cerebral hypoperfusion, it did not predict the presence of MCI in this study. Furthermore, when we controlled for age, the difference in cerebral PI between groups was lost. The MCI group was slightly older than the control participants and age did, but cerebral vascular outcomes did not, significantly discriminate between those with and without MCI in this study. Previous reports demonstrate that high cerebral PI (> 1.1) in adults with MCI leads to a higher risk ratio for accelerated cognitive dysfunction, and conversion to Alzheimer’s Disease (AD) (Lim et al. 2018, 2017). A possible explanation for the contrasting findings in our cohort might be due to the high individual variability in cerebral PI. Notably, when we treated cerebral PI as a categorical variable (i.e., cerebral PI > 1.1 = high) (Lim et al. 2018, 2017), our findings did not change.

Measures of cerebrovascular function were reasonably well-preserved in the MCI group. Besides resting MCAv not being affected, cerebrovascular CO_2_ reactivity was well maintained, and we also observed no detrimental impact of MCI on cerebral pressure-responsiveness (%∆MCAv%∆MAP). This is in line with the findings of De Heus et al. (2018), who reported a lower (better) %MCAv response for a given change in blood pressure in MCI compared to control, which is suggestive of maintained cerebral autoregulation in MCI. Our group (Klein et al. 2020) and others (Rosenberg et al. 2020) have recently suggested that higher arterial stiffness leads to the greater transmission of pulsatile blood velocity in healthy older adults. We observed that higher pressure-responsiveness in the control group was positively associated with systemic pressure augmentation (AIx) in this study. These findings raise the possibility that vascular impairment in adults with MCI is detectable only in the vulnerable segments of the systemic, but not cerebral circulation.

An increase in arterial pulse pressure is associated with stiffening and a reduced buffering capacity of the aorta and/or conduit arteries. Pulse wave velocity is widely used and considered the gold-standard non-invasive measure of aortic arterial stiffness (Townsend et al. 2015). We showed that adults with MCI have higher PWV compared with control participants. Central artery stiffening, including at the aorta, is associated with poor cognitive performance and structural brain changes such as white matter hyper-intensities and cerebral atrophy, in otherwise healthy older adults (Palta et al. 2019). Increased aortic stiffness reduces the pressure buffering capacity and may negatively impact cerebrovascular function through the transmission of excessive pressure pulsatility towards the cerebral circulation and microcirculation (Mitchell et al. 2011), although PWV and cerebral PI were not correlated in this study. High carotid artery pulsatility is associated with reduced global cerebral blood flow in MCI as measured by MRI (Tomoto et al. 2020). PWV has previously been associated with cerebral small vessel disease, Aβ-amyloid deposition and changes in cognition in adults with MCI (Hughes et al. 2018). Moreover, aortic stiffness is associated with reductions in regional cerebral perfusion, measured by magnetic resonance imaging, and cognitive scores in participants in the community-based Age, Gene/Environment Susceptibility–Reykjavik study who had no history of stroke, transient ischaemic attack or dementia (Jefferson et al. 2018).

Besides alterations in vascular structure, vascular endothelial function also likely plays a role in cognitive decline (Vendemiale et al. 2013a; Csipo et al. 2019). Impairments in vascular endothelial function have been reported in adults with AD (Dede et al. 2007; Venturelli et al. 2018), and among healthy older adults FMD was negatively associated with amyloid-β burden (Liu et al. 2019). In this study, we measured brachial artery FMD as an index of systemic endothelial function in adults with and without MCI. We observed reduced systemic vascular function in MCI, compared with control. It has recently been reported that there is little association between brachial artery FMD and cerebrovascular reactivity (Carr et al. 2020), which is consistent with our findings where cerebral CO_2_ reactivity was not altered in MCI compared with control. This might suggest that reduced systemic endothelial-dependent function is not related to altered cerebrovascular health in adults with MCI or that the mechanisms underpinning cerebral and systemic endothelial function differ (Ogoh and Bailey 2021). It has, however, been suggested that alterations in cerebrovascular reactivity with cognitive decline may only be evident as disease progresses longer-term, and following alterations in cerebral endothelial function (Jefferson et al. 2018). To examine this, future studies should aim to directly assess shear-mediated vasodilation of the extracranial arteries (Carter et al. 2016; Hoiland et al. 2017) as a direct assessment of cerebral endothelial function in adults with MCI.

High aortic stiffness (PWV) and endothelial dysfunction (FMD) predicted the presence of MCI in this study, independent of age. In contrast, cerebrovascular outcomes did not distinguish between MCI and healthy adults. Interestingly, when we accounted for age differences between the MCI and control groups, any differences in cerebrovascular function were lost. This is in line with previous findings showing no significant differences in cerebrovascular outcomes in healthy adults who are elderly (65 + years) and older-elderly (74 + years) (Oudegeest-Sander et al. 2014), or between adults with MCI and healthy adults of the same age (de Heus et al. 2018). Our findings suggest that the stronger combination of vascular assessments for detecting MCI are systemic in nature. These findings add to the growing body of evidence demonstrating the potential for systemic vascular biomarkers in the characterisation of cognitive decline. Our findings support the suggestion that systemic vascular dysfunction is an early feature of cognitive decline, which is not apparent in the cerebrovasculature when measured by TCD.

### Limitations

In the present study, cognitive status of healthy older adults and individuals with MCI was confirmed by using predefined MoCA cut-off scores (Nasreddine et al. 2005). Furthermore, individuals with MCI were recruited through the NeuroExercise project and had an established diagnosis of MCI according to the Albert et al. criteria (Devenney et al. 2017; Albert et al. 2011b). Even though the MoCA has high sensitivity (84%) and specificity (79%) to discriminate between healthy older adults and individuals with MCI (Roalf et al. 2013), it cannot fully exclude false positives and cannot be used to assess the severity of cognitive impairment. Therefore, future studies might apply a more extensive neuropsychological test battery, which would not only clarify the severity of cognitive impairment, but also allow for comparisons between different cognitive domains. Based on the screening procedures used it is unlikely that any of the control participants had dementia, although this possibility cannot be excluded as a clinical dementia rating assessment was not undertaken by the control group. Despite our attempts to recruit a healthy control group of similar age, we did have a small but significant difference (~ 3 years) between MCI and control in this study. However, both groups were over 65 years of age and all are considered “older adults”. Cerebrovascular outcomes have previously been shown to be similar between elderly (65–69 years) and older-elderly adults (74–86 years) (Oudegeest-Sander et al. 2014). There is evidence that cognitive impairment is more prevalent, and that it progresses at a faster rate in women compared with men (Lin et al. 2015; Sohn et al. 2018). The higher prevalence of cognitive impairment in women may be explained in-part by sex-related differences in cardiovascular risk factors and associated impairments in systemic and cerebrovascular function (Volgman et al. 2019). Our findings (see Online Resources, Supplementary Table 1) show that the differences in vascular function between people with and without MCI are likely to be generalisable to both males and females. We also found no differences between males and females for any of the measures of vascular and cerebrovascular function. However, this study was not powered for comparisons within or between the separate male and female cohorts and these comparisons should be verified in future studies with a larger sample size.

We did not measure physical activity levels or exercise capacity in all the individuals included in this study, and we cannot discount the possibility that potential differences in fitness levels between groups account for the differences in vascular outcomes we report. We have previously shown a positive association between cardiorespiratory fitness and MoCA score in adults with MCI (Stuckenschneider et al. 2018), and future research should aim to understand how cardiorespiratory fitness modifies cerebrovascular and systemic vascular function in adults with early cognitive decline. We deliberately excluded participants with prior cardiovascular events and untreated hypertension to maintain the homogeneity of the groups. It is recognised that cardiovascular disease exacerbates the risk of MCI and vascular dysfunction (Stefanidis et al. 2019, 2017). Based on the age and prescription-medication profile of the participants included in the present study, it is likely that some participants may have or are at risk of underlying cardiovascular disease. It was also apparent from some of the measures of spontaneous blood pressure, e.g. during the cerebrovascular assessments (see Supplementary Fig. 1), that blood pressure may have been poorly controlled in some participants, although mean blood pressure was not different between the MCI and CON groups. Future studies should confirm the present findings in those with and without a history of cardiovascular disease. To date, it is unclear which people with MCI progress to dementia, remain stable, or reverse to normal (Albert et al. 2011a; Roberts et al. 2014; Ganguli et al. 2011). Longitudinal prognostic studies are warranted to establish the role of cerebrovascular changes in the course of MCI and may help to identify which individuals are at the highest risk to progress to dementia. Consideration of intracranial cerebral blood flow dynamics is important for understanding cerebrovascular disease development. TCD is non-invasive and has high-temporal resolution, which allows for dynamic stimulus response measurement, and is often used in clinical populations. However, TCD does not measure changes in intracranial artery diameter, which have recently been shown to change during assessments where there are large changes arterial blood gases and blood pressure (Hoiland et al. 2019). Despite these limitations, TCD remains a very useful and reliable tool in the assessment of intracranial cerebral blood flow velocity.

## Conclusion

In the present study, cerebrovascular dysfunction, elevated cerebral pulsatility index and systemic arterial stiffening and endothelial dysfunction were observed in adults with mild cognitive impairment compared to health control participants. When accounting for age, only systemic vascular measures significantly distinguished between people with and without MCI. These findings provide further insight into the potential cardiovascular determinants of cognitive decline. Further research should focus on the detection and prevention of systemic vascular stiffening and endothelial dysfunction in adults at risk of cognitive decline.

## Supplementary Information

Below is the link to the electronic supplementary material.

Supplementary file1 (DOCX 610 KB)

## Abbreviations

AD: Alzheimer’s disease
AIx: Augmentation index
ANCOVA: Analysis of covariance
AP: Augmentation pressure
CBFv: Velocity of cerebral blood flow
cDBP: Central diastolic blood pressure
CO_2_: Carbon dioxide
cSBP: Central systolic blood pressure
CV: Coefficient of variation
CVC: Cerebrovascular conductance
FMD: Flow-mediated dilation
HR: Heart rate
MAP: Mean arterial blood pressure
MCAv: Middle cerebral artery blood flow velocity
MCI: Mild cognitive impairment
MoCA: Montreal cognitive assessment
Pb: Aortic backward pressure waveform
P_ET_CO_2_: End-tidal carbon dioxide
Pf: Aortic forward pressure waveform
PI: Cerebrovascular pulsatility index
PWV: Pulse wave velocity
RM: Reflection magnitude
TCD: Transcranial Doppler ultrasonography
