# Supplementary material for: Cerebrovascular function and its association with systemic artery function and stiffness in older adults with and without mild cognitive impairment
Source: Eur J Appl Physiol. 2022 May 6;122(8):1843–56. doi: 10.1007/s00421-022-04956-w (PMC9287231; doi:10.1007/s00421-022-04956-w)
Supplement: Supplementary file 1 — Supplementary file1 (DOCX 610 KB) [file 421_2022_4956_MOESM1_ESM.docx]

**ONLINE RESOURCES (supplementary materials)**

**Cerebrovascular function and its association with systemic artery function and stiffness in older adults with and without mild cognitive impairment**

Tom G. Bailey*^1,2^

Timo Klein*^1,3^

Annelise L. Meneses^1^

Kayla B. Stefanidis^1^

Stefanie Ruediger^2,3^

Daniel J. Green^4^

Tim Stuckenschneider^1,3^

Stefan Schneider^#1,3^

Christopher D. Askew^#1,5^

^1^ VasoActive Research Group, School of Health and Behavioural Sciences, University of the Sunshine Coast, Sippy Downs, QLD Australia.

^2^ Physiology and Ultrasound Laboratory in Science and Exercise, Centre for Research on Exercise, Physical Activity and Health, School of Human Movement and Nutrition Sciences, The University of Queensland, Brisbane, QLD, Australia.

^3^ Institute of Movement and Neurosciences, German Sport University, Cologne, Germany.

^4^ School of Human Sciences, Faculty of Science, The University of Western Australia, WA, Australia.

^5^ Sunshine Coast Health Institute, Sunshine Coast Hospital and Health Service, Birtinya, QLD, Australia.

*Joint first authors

^#^Shared senior author responsibilities

**Corresponding author:**

Associate Professor Christopher D. Askew, PhD

School of Health and Behavioural Sciences, University of the Sunshine Coast, 90 Sippy Downs Drive, Sippy Downs QLD 4556, Australia.

Telephone: +61 7 5456 5961. E-mail: [caskew@usc.edu.au](mailto:caskew@usc.edu.au)


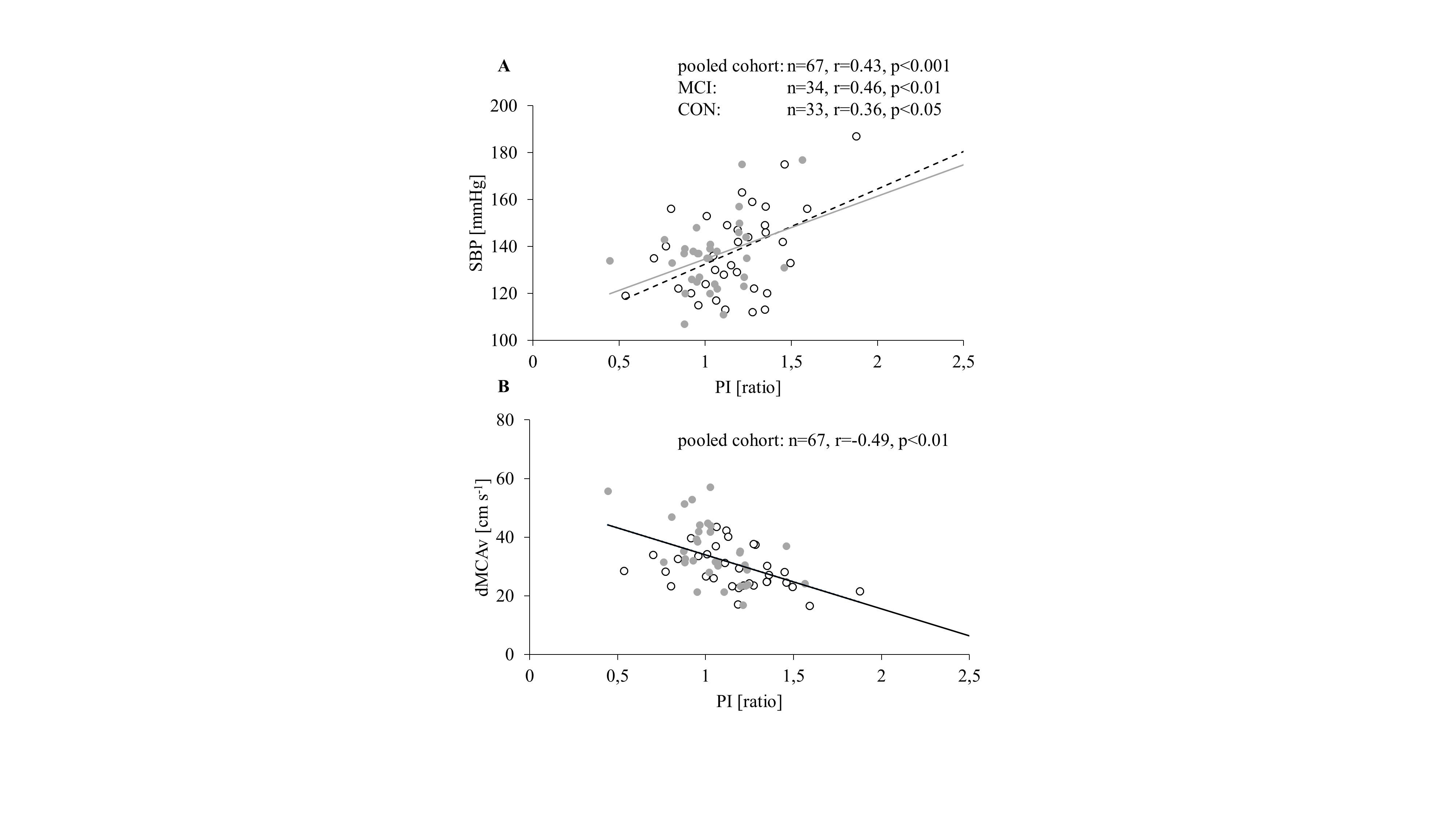


**Supplementary Figure 1**. Panel A: Correlation between systolic blood pressure (SBP) and cerebral pulsatility index (PI). Panel B: Correlation between diastolic middle cerebral artery velocity (dMCAv) and PI. Open circles are MCI and grey circles CON. The dashed line in A presents trend line for MCI and the grey line the trend for CON. Black line in B presents the trend for the full cohort.

**Supplementary Table 1. Cerebral and systemic vascular function measures in female and male participants**

| Variable | **Female** | | | **Male** | | |
| --- | --- | --- | --- | --- | --- | --- |
|  | MCI | CON | *p* value | MCI | CON | *p* value |
|  | Mean (95% CI) | Mean (95% CI) |  | Mean (95% CI) | Mean (95% CI) |  |
| MoCA (score) | 23.00 ± 2.13  (CI: 22.69-23.30); n=15 | 28.17 ± 1.38  (CI: 28.01-28.32); n=19 | **<0.001** | 22.30 ± 2.92  (CI: 22.08-22.52); n=26 | 27.50 ± 1.70  (CI: 27.26-27.73); n=14 | **<0.001** |
| **Cerebrovascular function measures** | | | | | | |
| MCAv supine rest (cm s^-1^) | 41.36 ± 9.66  (CI: 39.47-43.25); n=10 | 47.04 ± 14.17  (CI: 45.57-48.49); n=19 | 0.267 | 40.33 ± 11.04  (CI: 39.19-41.47); n=19 | 41.26 ± 9.35  (CI: 39.95-42.57); n=14 | 0.799 |
| CO2 reactivity –  (%cms^-1^ mmHg^-2^) | 2.92 ± 1.22  (CI: 2.67-3.15); n=10 | 2.79 ± 1.54  (CI: 2.63-2.94); n=19 | 0.826 | 2.15 ± 1.38  (CI: 2.02-2.29); n=20 | 2.71 ± 1.51  (CI: 2.50-2.92); n=14 | 0.273 |
| %MCA/%MAP (%%) | 1.10 ± 0.31  (CI: 1.04-1.15); n=11 | 1.60 ± 0.41  (CI: 1.55-1.64); n=19 | **0.001** | 1.33 ± 0.48  (CI: 1.29-1.37); n=23 | 1.37 ± 0.69  (CI: 1.27-1.46); n=14 | 0.865 |
| PI (ratio) | 1.17 ± 0.37  (CI: 1.10-1.24); n=11 | 0.99 ± 0.19  (CI: 0.97-1.01); n=19 | 0.087 | 1.16 ± 0.21  (CI: 1.14-1.18); n=23 | 1.10 ± 0.22  (CI: 1.07-1.13); n=14 | 0.400 |
| **Systemic vascular function measures** | | | | | | |
| FMD (%) | 4.74 ± 1.49  (CI: 4-68-5.20); n=13 | 5.83 ± 1.89  (CI: 5.81-6.23); n=19 | 0.128 | 4.08 ± 1.78  (CI: 4.03-4.31); n=26 | 4.50 ± 2.04  (CI: 4.32-4.92); n=14 | 0.496 |
| PWV (m.s^-1^) | 13.54 ± 2.47  (CI: 13.02-13.77); n=14 | 11.07 ± 3.08  (CI: 10.71-11.42); n=17 | **0.035** | 13.05 ± 2.17  (12.86-13.23); n=23 | 11.80  (11.57-12.03); n=13 | 0.075 |
| AIx75 (%) | 27.00 ± 7.49  (CI: 26.44-28.55); n=15 | 24.63 ± 7.70  (CI: 23.83-25.42); n=19 | 0.293 | 20.53 ± 11.86  (CI: 19.64-21.43); n=26 | 17.50 ± 9.57  (CI: 16.16-18.83); n=14 | 0.415 |
| SBP (mmHg) | 138.13 ± 19.09  (CI: 134.48-139.93); n=15 | 135.52 ± 13.52  (CI: 134.13-136.92); n=19 | 0.770 | 137.03 ± 17.99  (CI: 135.68-138.39); n=26 | 136.17 ± 17.25  (CI: 133.73-138.55); n=14 | 0.879 |
| DBP (mmHg) | 71.27 ± 8.61  (CI: 69.21-71.49); n=15 | 75.95 ± 10.09  (CI: 74.40-76.98); n=19 | 0.099 | 75.88 ± 10.12  (CI: 75.12-76.65); n=26 | 76.78 ± 9.59  (CI: 75.44-78.12); n=14 | 0.786 |

AIx, Augmentation index; DBP, diastolic blood pressure; MoCA, Montreal Cognitive Assessment, FMD, flow-mediated dilation; MCAv, middle cerebral artery flow velocity; PI, pulsatility index; PWV, pulse wave velocity; SBP, systolic blood pressure. MCI = mild cognitive impairment, CON = control.

**Supplementary Table 2. Correlations between cerebrovascular, systemic vascular outcomes and MoCA scores in MCI and control groups**

| Variable | **All** | | | **MCI** | | | **CON** | | |
| --- | --- | --- | --- | --- | --- | --- | --- | --- | --- |
|  | n | r | *p* | n | r | *p* | n | r | *p* |
| **Relationships between MoCA and systemic and cerebral measures** | | | | | | | | | |
| MoCA and FMD | 72 | 0.28 | **0.017** | 39 | 0.15 | 0.339 | 33 | 0.21 | 0.252 |
| MoCA and AIx | 74 | 0.01 | 0.947 | 41 | 0.08 | 0.606 | 33 | 0.17 | 0.351 |
| MoCA  and PWV | 67 | -0.22 | 0.068 | 37 | 0.17 | 0.314 | 30 | -0.03 | 0.864 |
| MoCA and MCAv (rest) | 62 | 0.29 | 0.018 | 29 | 0.25 | 0.174 | 33 | 0.16 | 0.358 |
| MoCA and %MCAv%MAP (stand-sit) | 67 | 0.23 | 0.059 | 34 | -0.15 | 0.428 | 33 | 0.39 | **0.026** |
| MoCA and %CO_2_reactivity | 63 | 0.06 | 0.601 | 30 | -0.16 | 0.392 | 33 | 0.03 | 0.849 |
| MoCA and PI | 67 | -0.19 | 0.114 | 34 | -0.02 | 0.897 | 33 | 0.04 | 0.826 |
| **Relationships between PI and systemic and cerebral measures** | | | | | | | | | |
| PI and FMD | 67 | 0.28 | **0.022** | 34 | 0.56 | **0.0001** | 33 | 0.23 | 0.19 |
| PI and Aix | 67 | -0.24 | 0.070 | 34 | -0.26 | 0.21 | 33 | -0.20 | 0.30 |
| PI and PWV | 62 | 0.16 | 0.21 | 32 | 0.06 | 0.72 | 30 | 0.14 | 0.46 |
| **Relationships between MCAv (rest) and systemic and cerebral measures** | | | | | | | | | |
| MCAv (rest) and FMD | 60 | 0.18 | 0.16 | 27 | 0.06 | 0.78 | 33 | 0.20 | 0.26 |
| MCAv (rest) and AIx | 62 | 0.16 | 0.23 | 29 | 0.22 | 0.28 | 33 | 0.09 | 0.63 |
| MCAv (rest) and PWV | 61 | 0.08 | 0.53 | 28 | 0.13 | 0.49 | 33 | 0.20 | 0.28 |
| **Relationships between %MCAv%MAP (stand-sit) and systemic and cerebral measures** | | | | | | | | | |
| %MCAv%MAP and FMD | 65 | 0.16 | 0.19 | 32 | -0.19 | 0.28 | 33 | 0.45 | 0.07 |
| %MCAv%MAP and AIx | 67 | 0.18 | 0.20 | 34 | 0.03 | 0.87 | 33 | 0.45 | **0.01** |
| %MCAv%MAP and PWV | 63 | 0.10 | 0.42 | 32 | 0.07 | 0.73 | 31 | 0.33 | 0.07 |
| **Relationships between %CO2reactivity and systemic and cerebral measures** | | | | | | | | | |
| %CO_2_reactivity and FMD | 61 | 0.08 | 0.51 | 28 | -0.12 | 0.54 | 33 | 0.16 | 0.36 |
| %CO_2_reactivity and AIx | 63 | 0.13 | 0.33 | 30 | 0.08 | 0.70 | 33 | 0.17 | 0.37 |
| %CO_2_reactivity and PWV | 59 | -0.02 | 0.84 | 28 | 0.09 | 0.63 | 31 | -0.05 | 0.77 |

AIx, Augmentation index; MoCA, Montreal Cognitive Assessment, FMD, flow-mediated dilation; MAP, mean arterial blood pressure; MCAv, middle cerebral artery flow velocity; PI, pulsatility index; PWV, pulse wave velocity. MCI = mild cognitive impairment, CON = control.
